# Remote sensing traffic scene retrieval based on learning control algorithm for robot multimodal sensing information fusion and human-machine interaction and collaboration

**DOI:** 10.3389/fnbot.2023.1267231

**Published:** 2023-10-11

**Authors:** Huiling Peng, Nianfeng Shi, Guoqiang Wang

**Affiliations:** School of Computer and Information Engineering, Luoyang Institute of Science and Technology, Luoyang, China

**Keywords:** remote sensing image processing (RSIP), multimodal sensing, robot multimodality, sensor integration and fusion, multimodal information fusion

## Abstract

In light of advancing socio-economic development and urban infrastructure, urban traffic congestion and accidents have become pressing issues. High-resolution remote sensing images are crucial for supporting urban geographic information systems (GIS), road planning, and vehicle navigation. Additionally, the emergence of robotics presents new possibilities for traffic management and road safety. This study introduces an innovative approach that combines attention mechanisms and robotic multimodal information fusion for retrieving traffic scenes from remote sensing images. Attention mechanisms focus on specific road and traffic features, reducing computation and enhancing detail capture. Graph neural algorithms improve scene retrieval accuracy. To achieve efficient traffic scene retrieval, a robot equipped with advanced sensing technology autonomously navigates urban environments, capturing high-accuracy, wide-coverage images. This facilitates comprehensive traffic databases and real-time traffic information retrieval for precise traffic management. Extensive experiments on large-scale remote sensing datasets demonstrate the feasibility and effectiveness of this approach. The integration of attention mechanisms, graph neural algorithms, and robotic multimodal information fusion enhances traffic scene retrieval, promising improved information extraction accuracy for more effective traffic management, road safety, and intelligent transportation systems. In conclusion, this interdisciplinary approach, combining attention mechanisms, graph neural algorithms, and robotic technology, represents significant progress in traffic scene retrieval from remote sensing images, with potential applications in traffic management, road safety, and urban planning.

## 1. Introduction

The global trend of urbanization stands as one of the prominent features of contemporary world development. Over time, an increasing number of people are flocking to urban areas in search of improved living conditions and broader opportunities. However, this trend exhibits marked differences across regions. In some areas, such as Europe and North America, urbanization has been a historical process spanning several centuries, resulting in relatively mature urban planning and infrastructure development (Chaudhuri et al., 2012). But, simultaneously, in some regions, especially in Asia and Africa, where development started later, the urbanization process is characterized by rapid growth, giving rise to a host of new challenges. This swift urbanization often leads to organizational issues stemming from inadequate urban planning, thereby impacting residents' quality of life and the sustainability of urban development. China, especially in a global context, serves as a noteworthy case study. As one of the most populous countries globally, China has undergone an unprecedented pace and scale of urbanization, profoundly influencing global urbanization trends. In this global context, the fusion of multi-modal information has become increasingly crucial for decision-making (Plummer et al., 2017). The integration of rapidly advancing remote sensing satellite technology with deep learning techniques has paved the way for significant advancements in various fields, including urban planning, disaster warning, and autonomous driving. The utilization of remote sensing data, acquired through spatial and spectral information obtained from digital image processing and analysis, has become an invaluable source for generating high-resolution satellite images (Liang et al., 2017). These images play a crucial role in tasks such as target extraction, map updates, and geographical information system (GIS) information extraction (Chaudhuri et al., 2012). However, extracting accurate road information from these high-resolution images has proven to be a challenging task using traditional methods that rely on grayscale feature analysis of image elements such as edge tracking or least squares B-spline curves. These methods suffer from issues related to accuracy, practicality, and generalizability. Therefore, this study aims to explore how to fully leverage remote sensing satellite technology and deep learning methods to address new challenges brought about by urbanization, especially in rapidly urbanizing regions like China.

To overcome these limitations, the integration of computer vision and deep learning (Wang et al., 2022; Wu et al., 2022, 2023; Zhang M. et al., 2022; Zhang Y.-H. et al., 2022; Chen et al., 2023) has become essential. Understanding the semantics of images has become increasingly important, and visual relationship detection has emerged as a key technique to improve computer understanding of images at a deeper level, providing high-level semantic information. In the early stages of visual relational research, common relationships between object pairs, such as position and size comparisons, were explored to improve object detection performance (Gaggioli et al., 2016). However, recent advancements have expanded the scope to include spatial object-object interactions, prepositional and comparative adjective relations, and human object interactions (HOIS) (Gaggioli et al., 2016), thereby enhancing the capabilities of vision tasks. Large-scale visual relationship detection has been achieved by decomposing the prediction of relationships into two parts: detecting objects and predicting predicates (Ben-Younes et al., 2019). Fusing various visual features, such as appearance, size, bounding boxes, and linguistic cues, has been employed to build base phrases, contributing to better phrase localization (Plummer et al., 2017). Furthermore, reinforcement learning frameworks and end-to-end systems have been proposed to enhance relationship detection through better object detection (Liang et al., 2017; Rabbi et al., 2020).

A combination of rich linguistic and visual representations has also been implemented using end-to-end deep neural networks, with the incorporation of external linguistic knowledge during training, leading to improved prediction and generalization (Kimura et al., 2007). Deep learning, proposed by Chander et al. (2009), has been a game-changer in many fields, surpassing traditional machine learning methods by automatically learning from large datasets and uncovering implicit properties in the data. Since its success in the ImageNET competition in 2012, deep learning has become widely adopted in computer vision, speech recognition, natural language processing, medical image processing, and remote sensing, producing state-of-the-art results. As a result, the integration of deep learning models in remote sensing has significantly improved various remote sensing-related tasks, driving advancements in the field as a whole. The combination of rapidly advancing remote sensing satellite technology with deep learning techniques has enabled more accurate and effective decision-making through multi-modal information fusion. This fusion has opened up new possibilities for a wide range of applications, from urban planning and disaster warning to unmanned driving and GIS updates, propelling the field of remote sensing and its integration with computer vision to new heights. However, compared to computer vision images, remote sensing images present more challenges due to their larger coverage area, wider variety of objects, and complex backgrounds. As a result, when extracting information from remote sensing images, it becomes essential to employ deep learning models with visual attention mechanisms to effectively identify relevant features amidst the complexity, leading to improved accuracy in information extraction. The visual attention mechanism is a concept inspired by the human brain's ability to filter out relevant information (Tang, 2022; Zheng et al., 2022) from vast visual input. By applying a global quick scan followed by a focus on specific regions, the visual attention mechanism helps humans efficiently process external visual information. Integrating this mechanism into deep learning models simulates the human visual system's way of handling external information, making it a significant aspect to study for enhancing automatic information extraction from ultra-high resolution remote sensing images.

**Figure 1:**
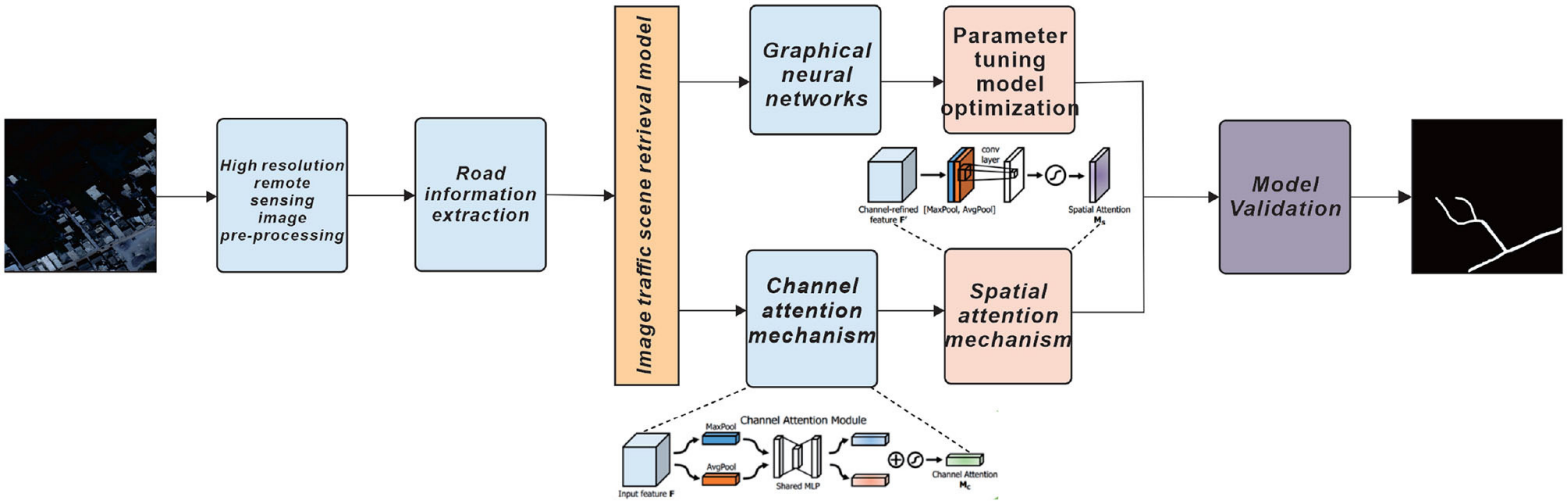
Schematic diagram of the overall structure of this paper, pre-processing of high-resolution remote sensing images, followed by extraction of road information from the images and model validation by combining Graph Neural Networks and dual attention mechanisms.

A more comprehensive elaboration of the research problem is indeed essential. The current introduction briefly mentions the challenges associated with traditional methods. Expanding on why these existing methods are inadequate, elucidating their limitations, and outlining why the integration of computer vision and deep learning is imperative will establish a robust foundation for this study (Liu et al., 2017). Traditional methods often rely on manual data collection and feature engineering, which can be labor-intensive, time-consuming, and susceptible to human error. These approaches may struggle to cope with the ever-increasing volumes of complex visual data generated in various fields. Moreover, traditional methods may not adapt well to dynamic environments or effectively handle subtle nuances and variations in data. In contrast, computer vision and deep learning techniques have demonstrated their ability to automatically extract meaningful features from raw data, recognize patterns, and adapt to changing scenarios. Their potential to revolutionize tasks such as image classification, object detection, and video analysis has made them indispensable in modern research and applications. By delving into the limitations of conventional methods and highlighting the advantages of integrating computer vision and deep learning, we establish a compelling rationale for the necessity of our study. In summary, this study aims to accurately extract road information from high-resolution remote sensing images by combining attention mechanism fusion and Graph Neural Networks. The proposed approach shows significant promise in enhancing information extraction (Gao et al., 2016; Liu et al., 2017) and ultimately contributing to more effective traffic management, safer roads, and intelligent transportation systems. The interdisciplinary nature of this research, linking remote sensing, robotics, and transportation, opens up new research possibilities and applications in traffic management, road safety, and urban planning (Figure 1 illustrates the overall structure of the research). The first part mainly introduces the development status of road information extraction based on remote sensing images, and describes the specific applications of road extraction methods at home and abroad, and lists the research objectives and significance of this paper, and explains the overall structure of this paper; the second part mainly introduces the related work, analyzes some of the most commonly used single pose estimation algorithms nowadays; the third part introduces the related algorithms used in this paper and the specific The fourth part describes the experimental process, which is based on the sample identification of the self-built dataset and the application of the Graph Neural Networks model algorithm with improved loss function, and verifies the validity and applicability of the model on the validation set, and compares some of the current mainstream algorithms. At the end of the paper, we discuss some advantages and disadvantages of the model, summarize the whole paper, and give an outlook on future work.

## 2. Related work

In the context of robotic multimodal information fusion decision-making (Luo et al., 2015), we can leverage advanced sensing technologies on robots to address the challenges in road extraction from remote sensing images (Mohd et al., 2022). Robots equipped with various sensors can efficiently acquire data and analyze images, enhancing the accuracy and speed of road network extraction compared to manual visual interpretation methods. To achieve automatic or semi-automatic extraction of road networks, we can integrate the dynamic programming algorithm into the robot's decision-making process (Kubelka et al., 2015). The dynamic programming algorithm, originally designed for low-resolution remote sensing images, has been improved for high-resolution images (Martins et al., 2015).

By incorporating this algorithm into the robot's capabilities, we can develop an efficient method for road feature extraction (Lin et al., 2020). The robot can use the dynamic programming algorithm to derive a parametric model of roads (Shi et al., 2023), treating it as a “cost” function and applying dynamic programming to determine the optimal path between seed points. This approach allows the robot to iteratively optimize the “cost” function, incorporating constraints specific to road features, such as edge characteristics, to enhance accuracy (Li et al., 2020). Moreover, the attention mechanism fusion can be applied to the robot's image analysis process. By integrating the attention mechanism into the extraction process, the robot can focus on specific road and traffic features, effectively reducing computation time while capturing relevant details for more accurate extraction results. Additionally, the use of graph neural algorithms can further enhance the robot's ability to detect and recognize road and traffic elements in the remote sensing images. These algorithms can process complex spatial relationships, improving the accuracy of scene retrieval.

With the robotic multimodal information fusion technique, the robot can autonomously navigate through urban environments and cover a wide area, capturing remote sensing images with high precision (Duan et al., 2022). This data acquisition capability will contribute to creating comprehensive traffic databases and supporting real-time traffic information retrieval for more accurate traffic management and planning. In conclusion, by combining the dynamic programming algorithm with attention mechanism fusion and graph neural algorithms, integrated into robotic multimodal information (Tang et al., 2021; He et al., 2023) fusion decision-making, we can significantly improve the efficiency and accuracy of road extraction from remotely sensed images. This interdisciplinary approach bridges remote sensing, robotics, and transportation, opening up new research opportunities and applications in traffic management, road safety, and urban planning.

In the context of our proposed approach for traffic scene retrieval from remotely sensed images, we can leverage the advantages of the Snake model and visual attention mechanism to enhance the effectiveness of feature extraction and improve the accuracy of information retrieval.

Firstly, the Snake model, with its feature extraction capabilities and energy minimization process, can be employed to accurately extract road contours and traffic elements from high-resolution remote sensing images. However, to overcome its sensitivity to initial position and convergence issues, we can integrate the Snake model with the attention mechanism fusion. By simulating the visual attention mechanism, we can quickly identify areas of interest in the input image, helping to place the Snake model near the relevant image features of roads and traffic elements, thus improving its efficiency and precision in extracting the target regions (Valgaerts et al., 2012). Moreover, the visual attention mechanism can play a crucial role in the process of compressing remote sensing image information. By analyzing human eye sensitivity to information, we can use different compression strategies based on the visual attention mechanism to compress remote sensing images effectively. This approach not only reduces the computational complexity but also improves the quality of the compressed images (Ghaffarian et al., 2021).

Additionally, for target detection in remote sensing images, we can utilize the visual attention computational model to calculate saliency maps of the images. By identifying regions of interest using these saliency maps, we can achieve accurate target recognition and classification, which is beneficial for traffic scene retrieval and understanding (Dong et al., 2021). Furthermore, semantic segmentation of remote sensing images can be improved by leveraging the visual attention mechanism. The attentional approach can assist in determining the exact boundaries of the targets to be extracted, leading to higher accuracy in information extraction from the remote sensing images (Buttar and Sachan, 2022). Overall, the combination of the Snake model and visual attention mechanism in the context of our proposed approach can significantly enhance traffic scene retrieval from remotely sensed images. By effectively capturing relevant details, reducing computational burden, and improving the accuracy of feature extraction, this interdisciplinary approach holds promise for revolutionizing traffic management, road safety, and urban planning through intelligent transportation systems. The integration of robotic multimodal information fusion decision-making will further extend the capabilities of this approach by enabling advanced sensing technologies and autonomous navigation for data acquisition and analysis in urban environments (Valgaerts et al., 2012; Dong et al., 2021; Ghaffarian et al., 2021; Buttar and Sachan, 2022).

In our research, we acknowledge the effectiveness of deep learning in high-resolution remote sensing image information extraction. Deep convolutional neural network models have shown promising results in extracting valuable information from very high-resolution (VHR) images. The use of Fully Convolutional Networks (FCNs) for semantic segmentation has become the mainstream approach in this domain. Various studies (Maggiori et al., 2017; Audebert et al., 2018; Bittner et al., 2018; Kampffmeyer et al., 2018; Li et al., 2018, 2023; Shahzad et al., 2018; Papadomanolaki et al., 2019; Razi et al., 2022) have demonstrated the benefits of employing deep learning methods to improve accuracy and feature representation in remote sensing image analysis.

Despite the progress made, the existing pixel-based methods can only reflect spectral information at an individual pixel level and lack a comprehensive understanding of the overall remote sensing image, leading to difficulties in obtaining meaningful object information and sensitivity to noise. Object-oriented and visual attention-based methods have shown potential but are limited by manual feature extraction and model robustness issues. Here, we propose a novel approach that incorporates attention mechanism fusion and robotic multimodal information fusion decision-making in the framework of graph neural algorithms to address these challenges (Chaib et al., 2022; Chen et al., 2022; Tian et al., 2023).

Our approach utilizes attention mechanisms to enhance the focus on specific road and traffic features in the remotely sensed images. By doing so, we effectively reduce parameter computation and improve the ability to capture relevant details. We further employ graph neural algorithms to enhance the accuracy of scene retrieval, enabling more precise detection and recognition of road and traffic elements. The integration of robotic multimodal information fusion brings a new dimension to the process. Robots equipped with advanced sensing technologies can autonomously navigate urban environments and capture high-accuracy, wide-coverage remotely sensed images. By leveraging this multimodal information, we create comprehensive traffic databases that facilitate real-time traffic information retrieval, contributing to more accurate traffic management and planning.

Through extensive experiments on large-scale remote sensing datasets, we demonstrate the feasibility and effectiveness of our proposed approach. The combination of attentional mechanism fusion, graph neural algorithms, and robotic multimodal information fusion enhances the retrieval of traffic scenes from remotely sensed images, resulting in improved accuracy and efficiency of information extraction (Wang et al., 2020; Tian and Ramdas, 2021). Ultimately, this leads to more effective traffic management, safer roads, and intelligent transportation systems (Chen et al., 2019; Cui et al., 2019; Li and Zhu, 2021). In conclusion, our interdisciplinary approach, which combines attentional mechanism fusion and robotic multimodal information fusion decision-making within the context of graph neural algorithms, presents significant advancements in retrieving traffic scenes from remotely sensed images. This integration of remote sensing, robotics, and transportation research opens up new possibilities for traffic management, road safety, and urban planning applications.

## 3. Method

### 3.1. Road feature extraction

The spatial features of roads in remote sensing images are mainly manifested as one or more narrow and long curves, while the differences between road information and other objects are mainly reflected in texture and spatial features. Based on such differences between different objects, the typical features of roads can be effectively extracted and input into Alex network for model training, and the typical features of roads in sample data can be derived. In this paper, three methods are mainly used to extract road features in remote sensing images, including rectangular matching degree, linear feature index and second-order rectangular features.

#### 3.1.1. Rectangular matching degree

Since the road is a two-way lane in the actual environment, the interval between the two curves is stable and constant under normal circumstances, they are parallel to each other with a short interval, which is expressed as two parallel lines in the remote sensing image. The parameter *M*_*R*_ in the rectangular matching degree in the road extraction is used as the feature parameter of the road object, this is for this feature of the road in the remote sensing image, *M*_*R*_ is expressed as:


(1) $$\begin{array}{l} M_{R}=\frac{X_{in}}{X_{oj}} \end{array}$$

Where: *X*_*in*_ indicates the area of the fitted rectangle of the image object; the larger the parameter *M*_*R*_ the more likely it is to be a road, and *vice versa*, the less likely it is to be a road; *X*_*oj*_ indicates the area of the acquired segmented image object.

#### 3.1.2. RecLinear characteristic index

It is difficult to express road features directly by spectral features in high resolution remote sensing images, traverse and calculate the minimum outer rectangle of all segmented image objects, and the rectangle satisfies:


(2) $$\begin{array}{l} L_{W}=N_{p} \end{array}$$

The linear characteristic index is obtained by calculating the centerline of all connected areas:


(3) $$\begin{array}{l} L_{W}=N_{p}I_{LF}=\frac{L}{W}=\frac{L}{\frac{N_{p}}{L}}=\frac{L^{2}}{N_{p}} \end{array}$$

Where, *N*_*P*_ denotes the area of the connected area; *I*_*LF*_ denotes the linear feature index; L and W denote the length and width of the image object; the larger the linear feature index, the more likely it is to be a road, and vice versa, the less likely it is to be a road.

#### 3.1.3. Second-order rectangular-like features

In high-resolution remote sensing images, for regular roads, using rectangular matching degree to express the feature parameters with linear feature indicators will work well, but like some complex roads similar to loops, it is more difficult to use rectangular matching degree to express the feature parameters with linear feature indicators. To address this problem, a new linear feature, i.e., the second-order rectangular feature, is invoked in this paper to describe complex shapes:


(4) $$C_{M}=\frac{\sum_{i=1}^{n}F_{i}}{n}$$

(5) $$\begin{array}{l} F_{i}=\sqrt{{\left(x_{i}-x_{m}\right)}^{2}+{\left(y_{i}-y_{m}\right)}^{2}} \end{array}$$

As shown in Equations (4) and (5) above, where *F*_*i*_ denotes the Euclidean distance between the ith image element and the center of mass inside the object; CM denotes the second-order moment characteristic parameter of the object; n denotes the number of image objects; i denotes the ith segmented object; *X*_*i*_, *Y*_*i*_ denote the arbitrary image element of the ith object; *X*_*m*_, *y*_*m*_, denote the center of mass position of the ith object in the row and column directions.

### 3.2. Graph Neural Networks

**Figure 2:**
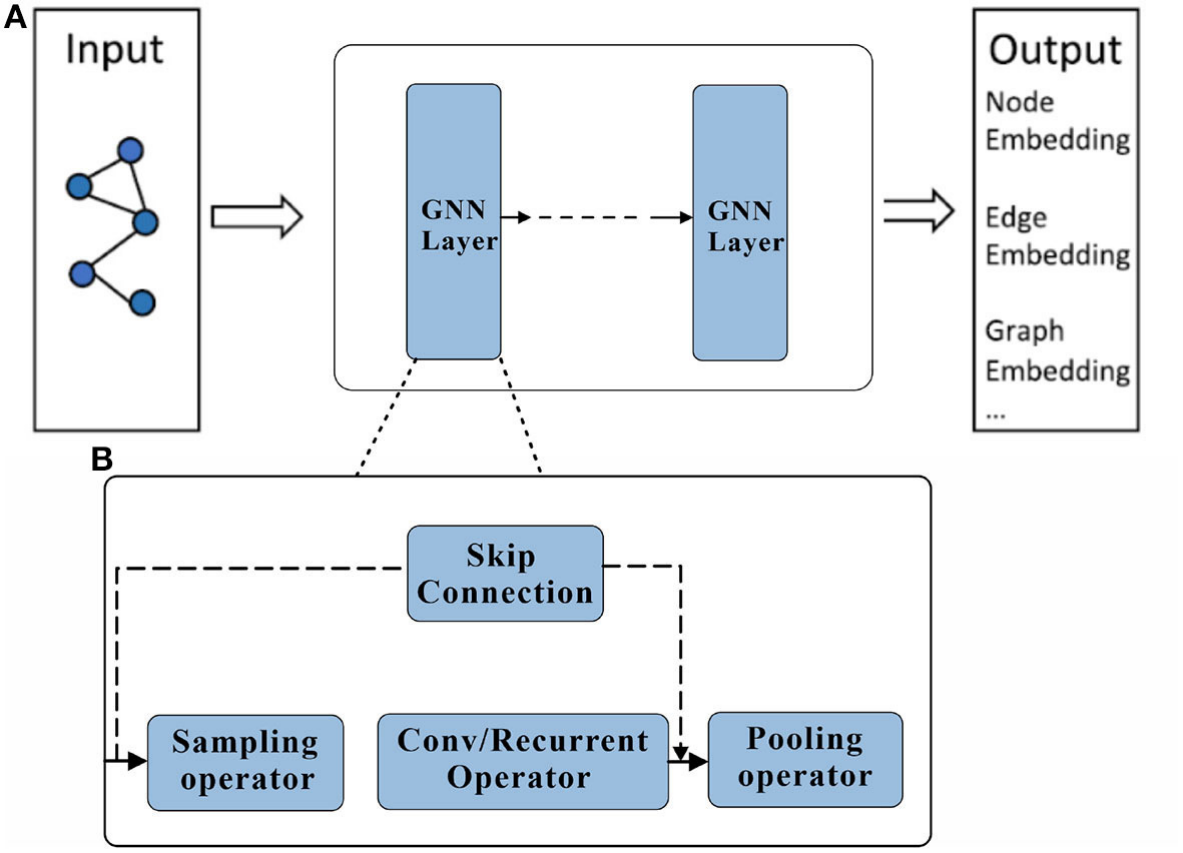
Graph Neural Network structure schematic. A Graph Neural Network consists of an input layer, an output layer and multiple hidden layers. %: **(A)** Description of what is contained in the first panel. **(B)** Description of what is contained in the second panel.

Graph Neural Networks (GNNs) are a type of deep learning model used for processing graph-structured data, and have achieved significant breakthroughs in fields such as image processing, social network analysis, and chemical molecule design. GNNs learn to represent the entire graph by iteratively propagating and aggregating information on the nodes and edges of the graph, enabling efficient processing of graph-structured data. The algorithmic principle of GNNs is as follows.The core idea of GNNs is to update the node representations through iterative information propagation and aggregation in the graph, starting from an initial feature representation for each node $x_{i}^{\left(0\right)}$ at time step *t* = 0, in an undirected graph *G* = (*V, E*) where *V* represents the set of nodes and *E* represents the set of edges. The update rule for each layer can be formalized as the following equation:


(6) $$\begin{array}{l} x_{i}^{\left(t+1\right)}=f\left(x_{i}^{\left(t\right)},{x_{j}^{\left(t\right)}}_{j\in N\left(i\right)},w^{\left(t\right)}\right) \end{array}$$

where $x_{i}^{\left(t\right)}$ represents the feature representation of node *v*_*i*_ at time step *t*, *N*(*i*) represents the set of neighboring nodes of node *v*_*i*_, *w*^(*t*)^ represents the parameter set at time step *t*, and *f*(·) represents the update function for node features. This update function usually consists of two parts: information aggregation and activation function. Information aggregation updates the feature representation of the current node by aggregating the features of its neighboring nodes, which can be achieved through simple weighted averaging or more complex attention mechanisms. The activation function introduces non-linearity to increase the expressiveness of the model. After multiple layers of information propagation and aggregation, the final node feature representation $x_{i}^{\left(T\right)}$ can be used for various tasks, such as node classification, graph classification, etc. For node classification tasks, the node features can be input into a fully connected layer followed by a softmax function for classification. For graph classification tasks, the features of all nodes are aggregated and input into a fully connected layer for classification, The structure of the Graph Neural Network is shown in Figure 2.

During the training process of GNNs, supervised learning methods are commonly used, optimizing the model parameters by minimizing the loss function between the predicted results and the true labels. The specific form of the loss function can vary depending on the task type and dataset, such as cross-entropy loss for node classification tasks, average pooling loss for graph classification tasks, etc. The algorithmic pseudo-code for the graphical convolutional neural network is shown in Algorithm 1.

**Algorithm 1:**
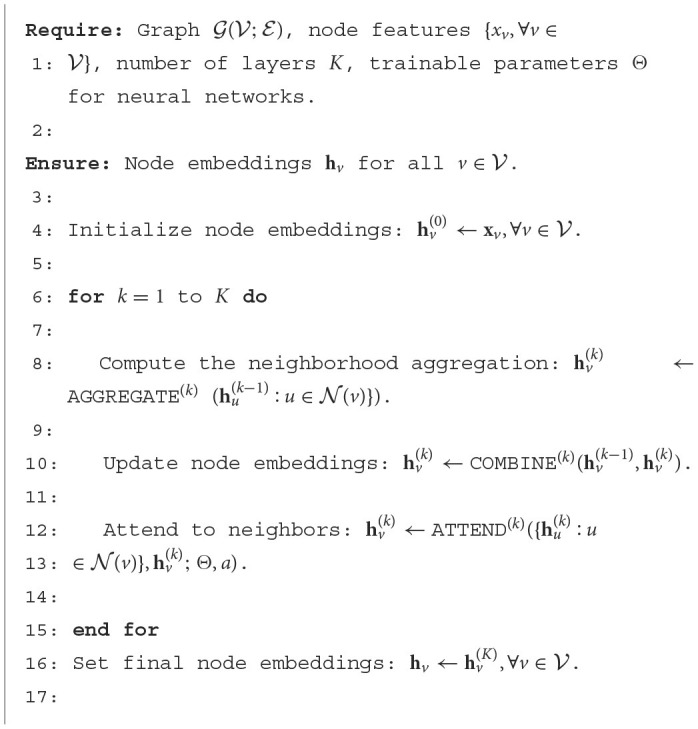
Graph Convolutional Neural Network (GCN).

In summary, GNNs learn to represent the entire graph by propagating and aggregating information on the nodes and edges of the graph, enabling efficient processing of graph-structured data.

### 3.3. Attention mechanism

Convolutional neural networks mimic the mechanism of biological visual perception, solving the tedious engineering of traditional manual feature extraction and realizing automatic feature extraction from data. However, the excellent performance of convolutional neural network is based on a large number of training samples, and the performance of convolutional neural network decreases dramatically under small samples because the training data is small and the sample features cannot be extracted comprehensively and effectively, so the attention mechanism is introduced into the convolutional neural network.

**Figure 3:**
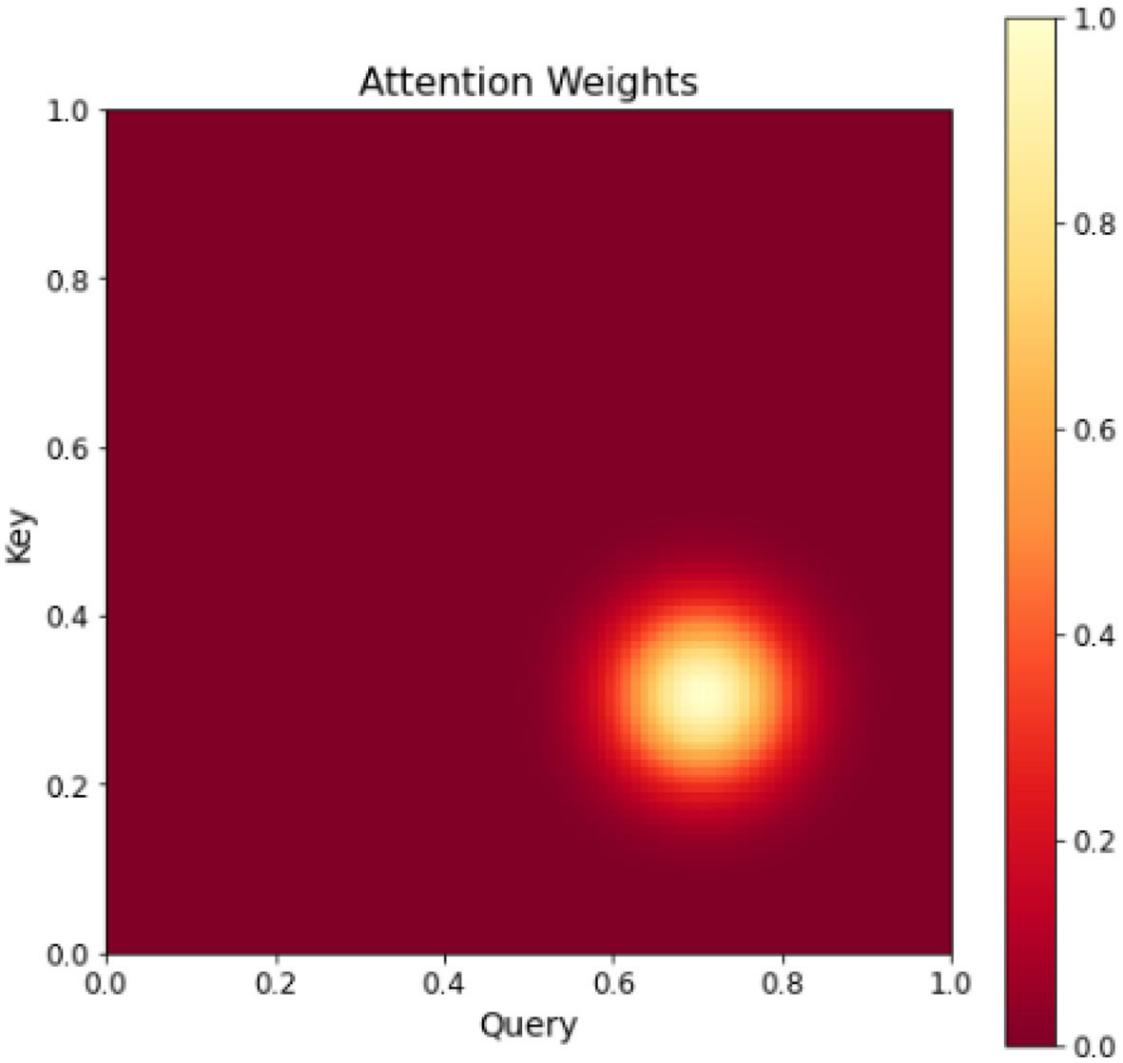
Heat map of attention mechanism.

Visual attention mechanism as an important feature of human visual system. Combining attention mechanism with deep learning models, attention mechanism can help deep learning models to better understand external information. In deep learning attention mechanism is an effective tool to extract the most useful information from the input signal. Attention mechanisms usually use higher-level semantic information to reweight lower-level information to suppress background and noise. The attention mechanism is implemented by using filtering functions (e.g., softmax and sigmoid) and sequential techniques. Attention mechanisms combined with deep learning models have been applied to target detection, natural language processing, image classification, semantic segmentation, etc. with some success. Figure 3 displays a more complex attention mechanism, represented by a two-dimensional matrix that depicts the attention weights between Query and Key pairs. Each element in the matrix represents the attention weight between a Query and Key pair. The darker colors indicate higher attention weights, while lighter colors indicate lower attention weights.

The attention mechanism mimics the human visual attention pattern, focusing on only the most relevant originating information to the current task at a time, making the information request more efficient. The combination of attention mechanism and convolutional neural network can make the network model pay more attention to important features, increase the weight of important features, and suppress unnecessary features to further improve the feature extraction ability of convolutional neural network for important information. In this paper, a dual attention mechanism combining channel attention mechanism and spatial attention mechanism is introduced to combine Graph Neural Networks with a custom sample data set for more accurate detection and recognition of roads and transportation tools.

#### 3.3.1. Channel attention mechanism

The channel attention mechanism is based on a basic understanding of convolutional neural networks: features of different parts of an object are encoded on different channels of the convolutional feature map. The basic idea of the channel attention mechanism is to continuously adjust the weights of each channel through learning, and generate a vector of length equal to the number of channels through the network, and each element in the vector corresponds to the weight of each channel of the feature map, which in essence tells the network the parts of the pedestrian to be attended to. An attention mechanism algorithm pseudo-code is shown in Algorithm 2.

**Algorithm 2:**
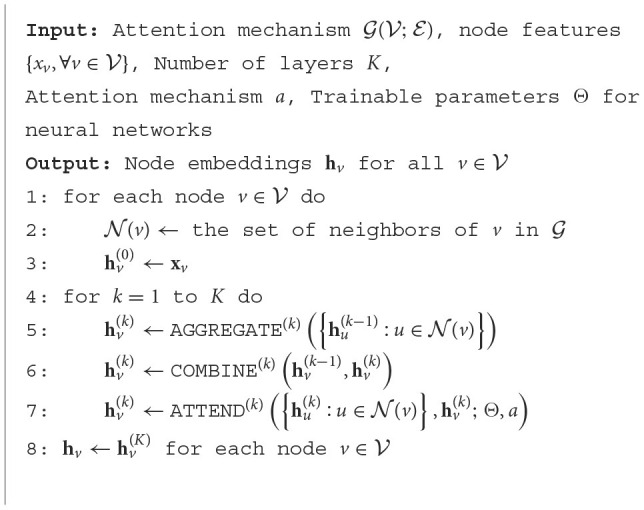
The attention mechanism algorithm pseudo-code.

**Figure 4:**
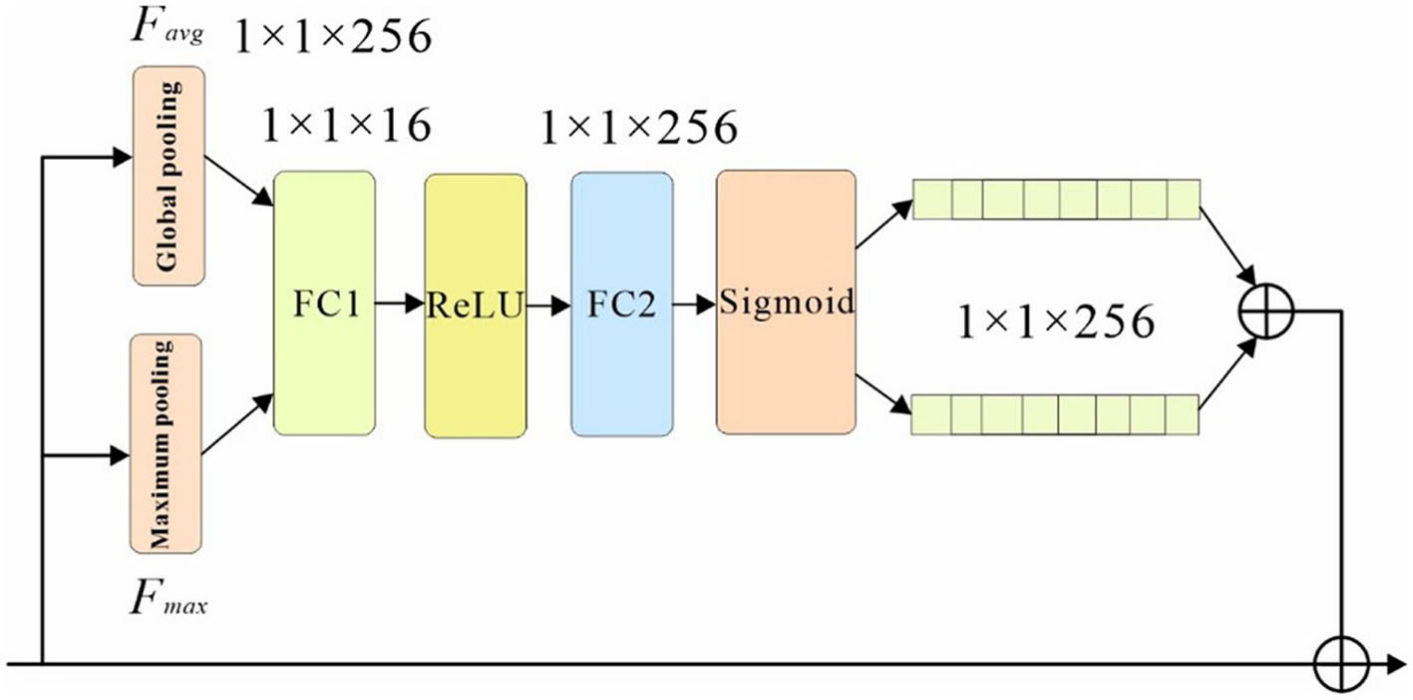
Channel attention sub-network structure.

The network structure is shown in Figure 4, where the classification branches are pooled first; the pooled weight vectors are fed into the fully connected layers FC1 and FC2 for “compression” and “stretching” operations. Then the components of the vectors are restricted between 0 and 1 by the sigmoid function, and the two vectors are summed and fused to form the final weight vector. In this paper, global pooling and maximum pooling are used simultaneously to highlight the main features while preserving the average characteristics of each channel, allowing the network to pay more attention to the visible parts of the pedestrians. The channel attention module generates a channel attention map using inter-channel relationships between features, and this feature assigns greater weight to channels where salient targets exhibit high response, as shown in the schematic diagram of the channel as attention module structure in Figure 4.

First, the input feature F is subjected to both maximum pooling and average pooling operations, and the null of the aggregated feature mapping The interval information is then input to a shared network, and the spatial dimension of the input feature map is compressed to sum the elements in the feature map one by one and generate the channel attention weights. The calculation formula is shown in equation below.


(7) $$\begin{array}{l} L_{r}\left(t,t^{*}\right)=\underset{n\in A}{\sum }\left(p_{n}^{*}=1\right)\underset{i\in x,y,w,h}{\sum }smoothL1\left(t_{i}^{n}-t_{i}^{*n}\right) \end{array}$$

#### 3.3.2. Spatial attention mechanism

**Figure 5:**
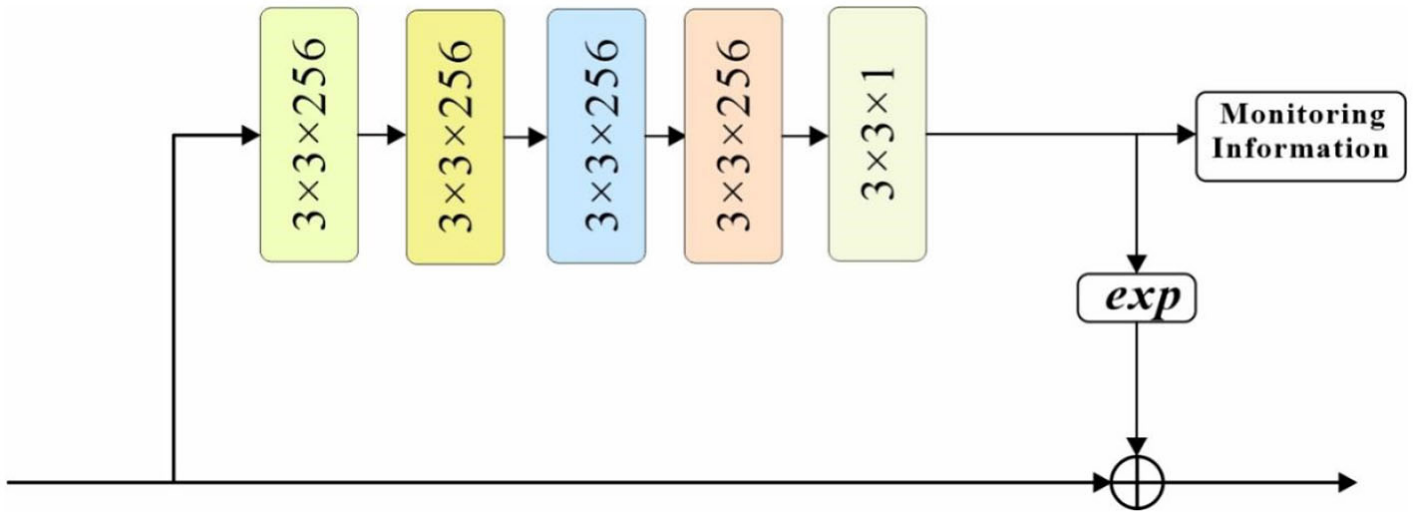
Spatial attention sub-network structure.

Another attention mechanism cited in this paper is the spatial attention mechanism, which is essentially a network structure that generates a mask of the same size as the original image features, where the value of each element in the mask represents the feature map weight corresponding to the pixel at that location, and the weights change as they are continuously learned and adjusted, which in essence tells the network which regions to focus on. As shown in Figure 5, the sub-network structure of the spatial attention mechanism in this paper.

The feature map is first convolved by four 3 × 3-sized convolutional checks and return branches with both 256 channels, and then compressed into a single mask with 3 × 3 convolution and 1 channels. the original feature map is multiplied by EXP (mask parameter) multiplied to retain the original background information, thus adjusting the weights of each position of the original feature map. To guide the learning of the spatial attention mechanism, this paper uses the supervised information of the pedestrian as the label of the spatial attention mechanism to generate a pixel-level target mask: the pixel values of the pedestrian's full-body bounding box and visible bounding box regions are set to 0.8 and 1, respectively, and the pixel values of the remaining background regions are set to 0. Such a labeling will guide the spatial attention mechanism to focus its attention on the road regions in the frame at the The spatial attention mechanism is guided to pay more attention to the road visible area while focusing on the road area in the picture.

### 3.4. Loss function

The loss function is an important part of the deep learning process, and the main purpose of using the loss function in this paper is to evaluate the prediction accuracy of the model and adjust the weight turnover, the loss function can make the convergence speed of the neural network become faster and make the prediction accuracy of the traffic scene more accurate. The most obvious difference between the two types of problems is that the result of regression prediction is continuous (e.g., house price), while the result of classification prediction is discrete (e.g., handwriting recognition). For example, semantic segmentation can be thought of as classifying each pixel in an image, and target detection can be thought of as regression on the position and size of a Bounding Box in an image.

#### 3.4.1. Overall loss function of the algorithm

In this paper, the parameters of each component are tuned jointly by a multi-task loss function, which consists of 3 components.


(8) $$\begin{array}{l} Loss=\frac{1}{M^{c}}\underset{n\in A}{\sum }L_{c}\left(p_{n},p_{n}^{*}\right)+\lambda_{1}\frac{1}{M^{r}}L_{r}\left(t,t^{*}\right)+\lambda_{2}L_{a}\left(m,m^{*}\right) \end{array}$$

where: $L_{c}\left(p_{n},p_{n}^{*}\right)$ is an improved classification loss function in the basic form of a weighted cross−entropy loss function, whose main purpose is to improve the problem of extreme imbalance between positive and negative samples in the regression−based traffic scene detection algorithm; *M*^*c*^ is the number of all predicted frames; *M*^*r*^ is the number of all predicted frames, considering only the part judged as foreground; $L_{a}\left(m,m^{*}\right)$ is the loss function of the spatial attention mechanism sub-network, which *L*(m, m^*^) is the loss function of the spatial attention mechanism subnetwork, which is actually a cross−entropy loss function based on each pixel of the mask; p and p denote the category probability of the nth predicted traffic frame and the corresponding actual category, respectively; $L_{r}\left(t,t^{*}\right)$ is a new regression loss function proposed in this paper, which can design the size of the weights independently according to different masking degrees, and its design ideas and details will be introduced below; m, m^*^ are the masks generated by the spatial attention mechanism and their corresponding mask labels, respectively; λ_*m*_, *m*_1_ are the masks generated by the spatial attention mechanism and their corresponding mask labels; λ and λ_2_ are the parameters used to balance the sub−loss functions, and their values are both 1 in this paper.

#### 3.4.2. Regression loss function for occlusion perception

In generic target detection, the classical regression loss function is the smooth L1 function, which takes the form:


(9) $$\begin{array}{l} L_{r}\left(t,t^{*}\right)=\underset{n\in A}{\sum }\left(p_{n}^{*}=1\right)\underset{i\in x,y,w,h}{\sum }smoothL1\left(t_{i}^{n}-t_{i}^{*n}\right) \end{array}$$

The expected weight is between 0 and 1, and even for a perfectly correct prediction frame, its IOU with the visible area may be a smaller value. The overlap between the visible area of the road and the prediction frame is used to improve the occlusion problem, and the practice is to judge this prediction frame as a positive sample only when the IOU of the prediction frame with both the road boundary frame and the visible area boundary frame is greater than a fixed threshold.

## 4. Experiment

### 4.1. Experimental data set

**Figure 6:**
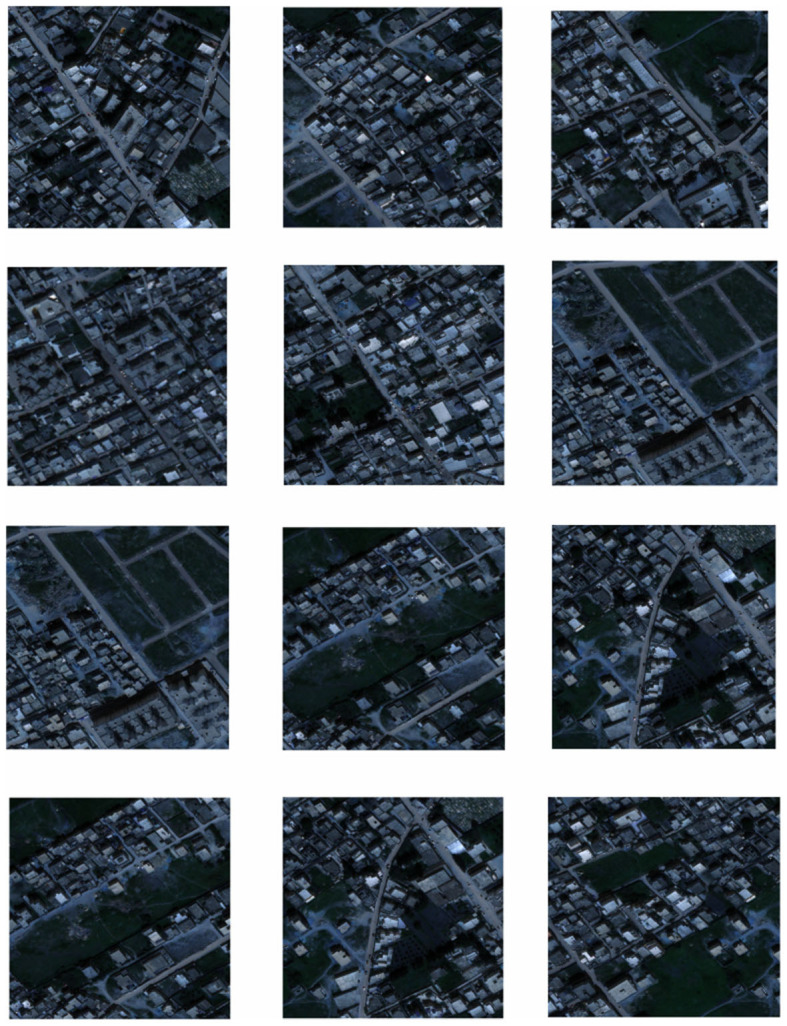
Remote sensing image map in the dataset.

**Figure 7:**
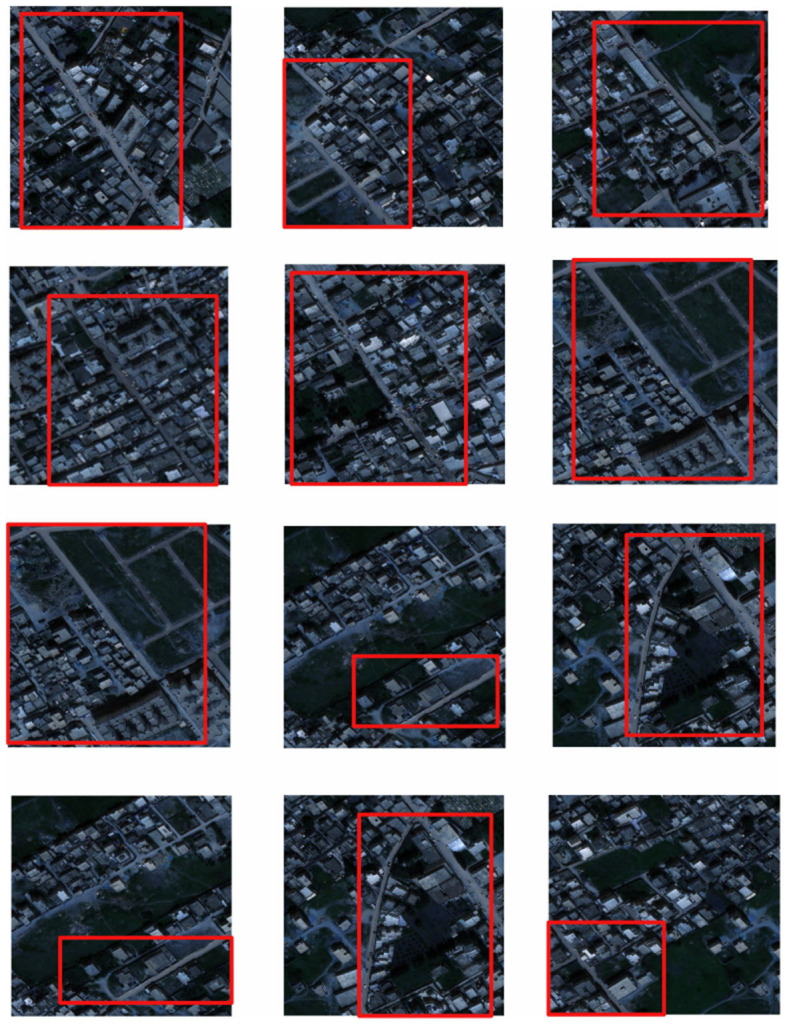
Target extraction area in remote sensing images.

The AI-TOD aerial image dataset was selected for training. 700,621 object instances in 8 categories from 28,036 aerial images were included in AI-TOD. In addition, 1000 instances of data from the Inria aerial image dataset were used as the validation set for model validation, As shown in Figure 6. The dataset consists of 1171 3-channel images and corresponding 2-channel segmentation labels with a spatial resolution of 1m, and each image has a size of 1500*1500 pixels. The labels are binarized images with a road pixel value of 1 and a background pixel value of 0. The dataset is randomly divided into three groups,seventy percent for the training set, twenty percent for the test set, and ten percent for the validation set. To avoid overfitting, the image preprocessing was first performed using a sliding window cropping technique with a span of 256 pixels, As shown in Figure 7; then standard data enhancement was performed, and all cropped images were cropped, scaled, randomly rotated, horizontally and vertically flipped and image color changed. Some of the example data in the dataset are shown below.

### 4.2. Experimental platform

This paper uses Pytorch 1.10.0 deep learning framework, the operating system environment is Windows 10, the GPU is NVIDIA GeForce GTX 1650, the system memory size is 24G, and the programming language is Python 3.8.0. The initial learning rate is 0.001, which decreases to 0.0001 at 200 rounds and decays to 0.00001 at 260 rounds. 260 rounds of network training are conducted, and the batchsize of each GPU is 32. The images are modified by image preprocessing. In this paper, we crop the images in the COCO dataset to 256 × 192 size, and then achieve the data enhancement effect by random flipping and random scaling.

### 4.3. Evaluation criteria

Remote sensing images are widely used in various fields such as urban planning, traffic management, and environmental monitoring. Road extraction from remote sensing images is an essential task that enables the identification and mapping of transportation networks. It helps in building precise and accurate geographic information systems and enhancing the performance of autonomous vehicles. However, the automatic road extraction methods face significant challenges due to the complex and diverse environments of remote sensing images. Therefore, it is necessary to evaluate the quality of the road extraction methods using appropriate metrics. In this context, recall rate and cross-merge ratio are universal evaluation metrics that are commonly used to assess the performance of remote sensing image road extraction methods. The recall rate is defined as the ratio of true positive predictions to the total number of actual positive samples, which represents the ability of the method to detect all the road pixels. It is given by the following formula:


(10) $$\begin{array}{l} R=\frac{TP}{TP+FN} \end{array}$$

Where TP is the number of true positives, and FN is the number of false negatives.

On the other hand, the cross-merge ratio represents the accuracy of the method in identifying the actual road pixels. It is defined as the ratio of true positive predictions to the total number of predicted road pixels, including both true positives and false positives. It is given by the following formula:


(11) $$\begin{array}{l} U=\frac{TP}{TP+FP} \end{array}$$

Where FP is the number of false positives.

Both recall rate and cross-merge ratio are important metrics for evaluating the performance of road extraction methods. However, they have their limitations. For example, the recall rate only considers the ability of the method to detect road pixels, but it does not measure the accuracy of the detection. Therefore, a method with a high recall rate may produce a large number of false positives. On the other hand, the cross-merge ratio only measures the accuracy of the predicted road pixels, but it does not consider the ability of the method to detect all the road pixels. Therefore, a method with a high cross-merge ratio may miss some road pixels.

To overcome these limitations, other evaluation metrics have been proposed, such as F1-score, precision, and accuracy. The F1-score is the harmonic mean of precision and recall, and it is given by the following formula:


(12) $$\begin{array}{l} F1=\frac{2PR}{P+R} \end{array}$$

Where P is the precision, which is the ratio of true positive predictions to the total number of predicted positive samples, and it is given by the following formula:


(13) $$\begin{array}{l} P=\frac{TP}{TP+FP} \end{array}$$

The accuracy is the ratio of the total number of correct predictions to the total number of predictions, and it is given by the following formula:


(14) $$\begin{array}{l} accuracy=\frac{TP+TN}{TP+TN+FP+FN} \end{array}$$

### 4.4. Analysis of experimental results

**Figure 8:**
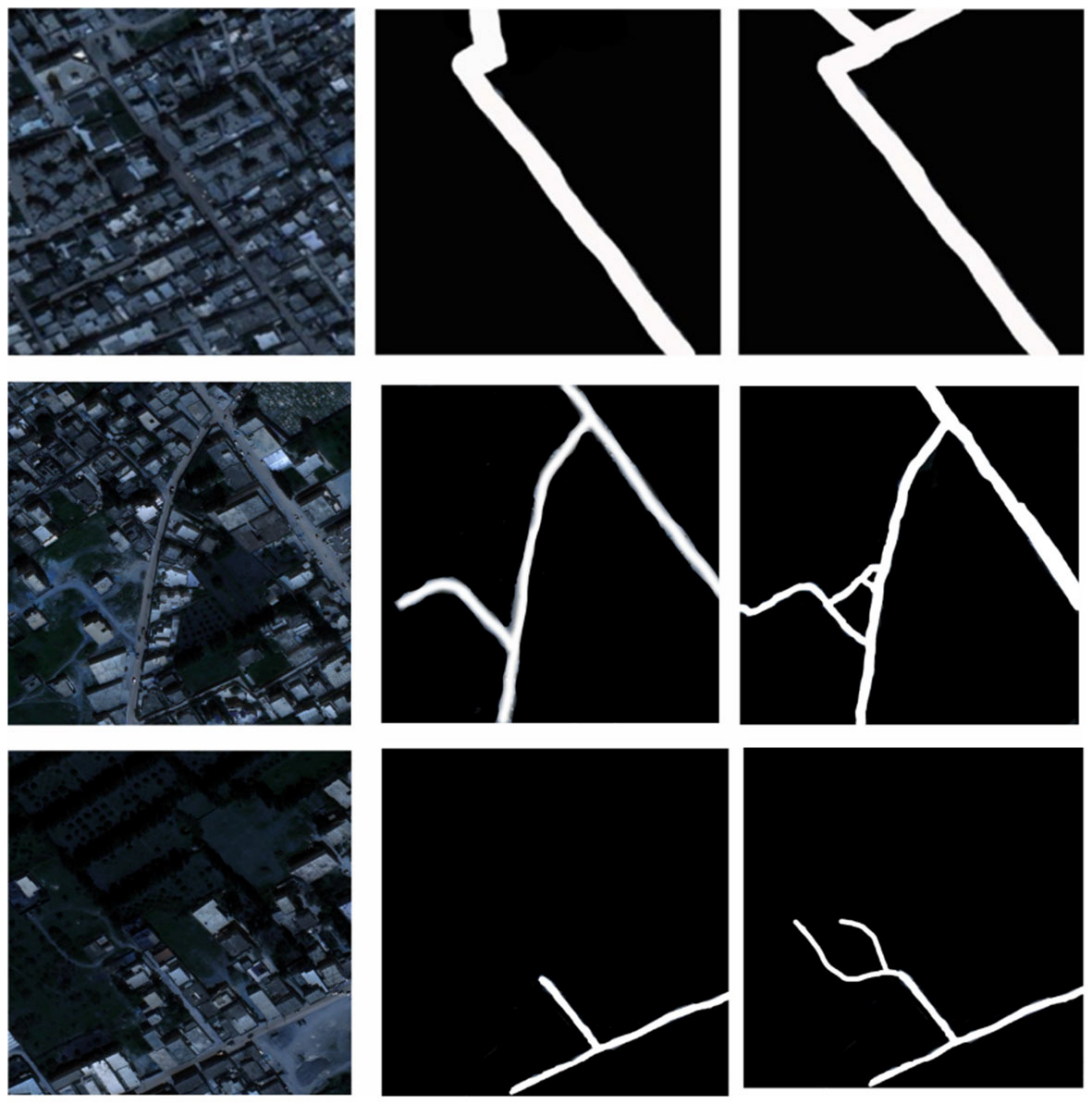
Results of model application in real remote sensing road extraction.

**Figure 9:**
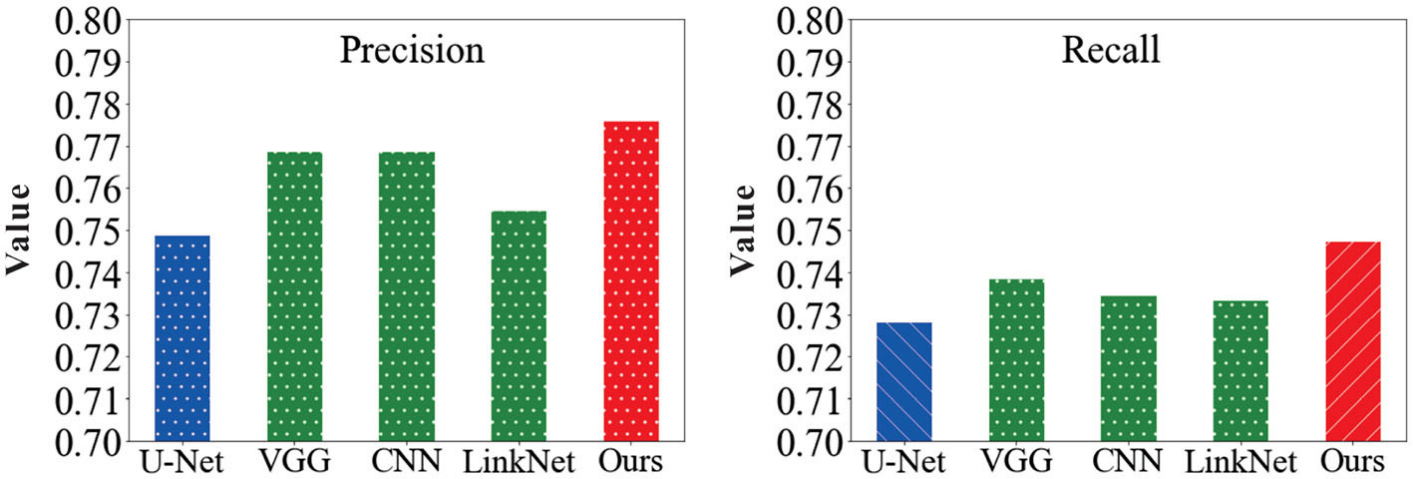
Comparison of precision and recall results values of the model in this paper with other network models.

**Figure 10:**
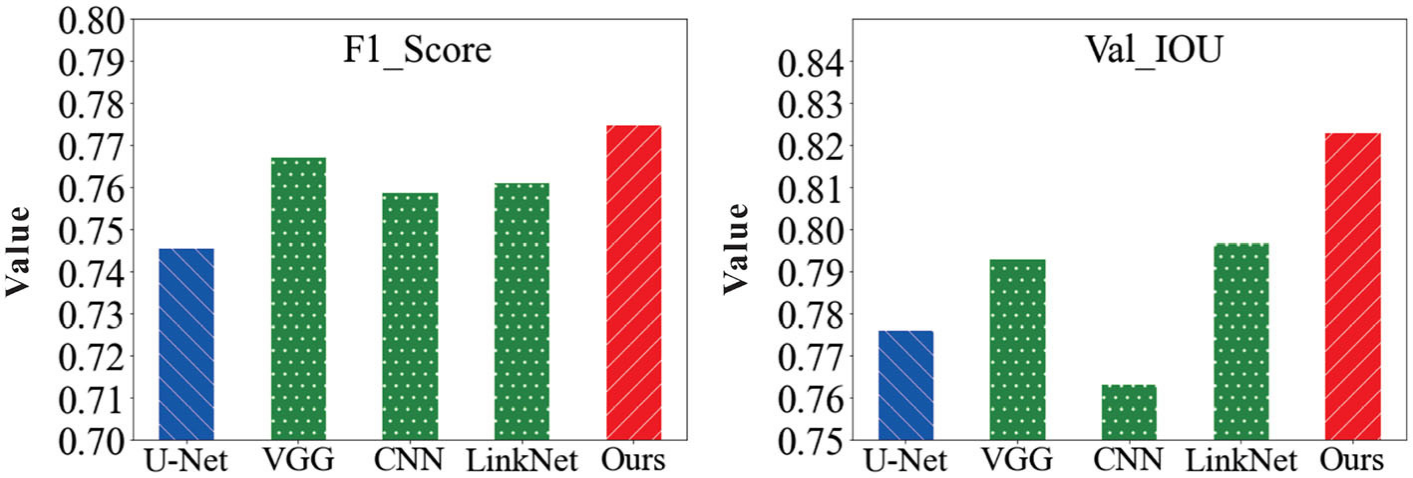
Comparison of F1-score and Val-IOU results values of the model in this paper with other network models.

In order to verify the feasibility of the road segmentation model, the U-Net model based on the U-Net model and the improved model in this paper to implement the road-road extraction task for high-resolution remote sensing images, As shown in the Figure 8. Among them, the parameters of the comparison network are set the same as the original method. The performance comparison of different models for road segmentation is given in Table 1. It can be seen in Figures 9, 10 that: the recall rate of this model is improved by five percent compared with that of U-Net, which is more consistent with the real labels and has better recognition rate for roads in image. the cross-merge ratio of this model is improved by zero point eight percent compared with that of U-Net, It shows the superior performance of the model in road extraction.

**Table 1:** Results of experimental models compared to other research models.

**Model**	**Accuracy**	**Recall**	**Precision**	**F1-score**	**Val-IOU**
U-Net (Shahzad et al., 2018)	80.1	73.1	74.8	74.3	77.6
VGG (Kampffmeyer et al., 2018)	76.5	74.12	76.89	77.05	79.23
CNN (Razi et al., 2022)	83.1	73.25	76.85	75.66	76.21
DCNN (Razi et al., 2022)	84.2	75.21	77.82	76.23	77.85
LinkNet (Papadomanolaki et al., 2019)	84.51	73.57	75.61	76.23	79.44
D-LinkNet (Papadomanolaki et al., 2019)	85.2	74.02	76.13	76.87	80.32
Ours	87.13	75.68	77.34	77.17	82.23

In order to compare the performance of different attention modules, this paper introduces several attention modules into GNN. The results are shown in Table 2. Except for the ECA attention module, all the other three attention modules gained in the U-Net network and achieved better performance than the baseline network. Better performance than the baseline network was achieved.

**Table 2:** U-Net comparison experiments with several different attention modules.

**Method**	**Recall**	**Precision**	**F1-score**	**Val-IOU**
ECA-GNN (Shahzad et al., 2018)	75.24	75.53	73.45	76.3
GNN (Kampffmeyer et al., 2018)	76.91	74.23	74.57	77.13
CBAM-GNN (Razi et al., 2022)	77.31	73.23	75.21	76.88
SE-GNN (Razi et al., 2022)	75.48	76.58	75.74	76.93
GC-GNN-Net (Razi et al., 2022)	77.82	74.75	74.89	76.65
Ours	78.49	75.85	76.43	77.98

## 5. Discussion

In response to the challenges faced in deep learning-based high-resolution remote sensing image information extraction, there are several issues that need to be addressed, such as the lack of global contextual information, over-segmentation problems, and difficulties in fusion of multimodal data. Additionally, the computational complexity, long training periods, and limited information transfer in both vertical and horizontal directions pose further challenges. To overcome these issues and improve the accuracy of remote sensing image information extraction, our proposed approach that integrates global attention mechanisms and robot-assisted techniques holds great promise in addressing the challenges of deep learning-based high-resolution remote sensing image information extraction. By incorporating global attention, we enhance the perception of scenes, improve object classification accuracy, and overcome the lack of global contextual information. Furthermore, the spatial attention mechanism helps reduce over-segmentation problems and facilitates the fusion of multimodal data, resulting in more accurate and detailed feature extraction. Robots equipped with advanced sensors and cameras are invaluable in the remote sensing domain. Their capabilities in data acquisition, preprocessing, and feature extraction contribute significantly to improving the efficiency and accuracy of remote sensing image analysis. Moreover, robot assistance in data fusion and annotation reduces manual efforts and enhances overall extraction processes.

The interdisciplinary nature of our approach bridges the gap between remote sensing, robotics, and transportation, opening new possibilities for research and applications in traffic management, road safety, and urban planning. By leveraging the strengths of attention mechanisms and robot-assisted techniques, we can achieve enhanced information extraction, leading to more effective traffic management, safer roads, and intelligent transportation systems. This advancement in the field of high-resolution remote sensing image analysis and robotics will undoubtedly have important economic value and far-reaching research significance in various applications and industries.

## 6. Conclusion

In the context of robotics, the proposed approach holds significant potential to augment the capabilities of robotic systems in road and transportation management. By integrating remote sensing and deep learning technologies into robots, they can actively contribute to various tasks, including road monitoring, traffic flow analysis, and autonomous navigation. With the ability to extract road and transportation features from remote sensing images, robots can efficiently carry out tasks such as road inspection, traffic surveillance, and swift response to accidents. The enhanced retrieval of traffic scenes from remotely sensed images, facilitated by the attentional mechanism fusion and graph neural algorithms, provides robots with precise and up-to-date information for effective decision-making in real-time traffic management and planning.

This integration of robotics with remote sensing and deep learning not only improves the efficiency of traffic-related tasks but also enhances road safety and overall transportation systems. With robots capable of autonomously navigating urban environments and capturing high-resolution remotely sensed images, comprehensive traffic databases can be created, allowing for more accurate and informed traffic management strategies.

As robotics technology continues to advance, the potential for further advancements in the field of intelligent transportation becomes even more promising. The ongoing evolution of deep learning techniques and visual attention mechanisms will continue to shape the future of remote sensing information extraction, enabling robots to play an even more significant role in traffic management, road safety, and urban planning. However, it is essential to acknowledge that current remote sensing information extraction methods based on deep learning still heavily rely on large training datasets, which can be resource-intensive to produce. Future research should focus on addressing this challenge and finding innovative ways to integrate domain knowledge and manual expertise with deep learning models to reduce dependence on extensive sample sets. By combining human expertise with cutting-edge technologies, the potential for advancing intelligent transportation systems becomes even greater.

## Data availability statement

The original contributions presented in the study are included in the article/supplementary material, further inquiries can be directed to the corresponding author.

## Author contributions

HP: Conceptualization, Data curation, Formal analysis, Investigation, Methodology, Project administration, Resources, Supervision, Visualization, Writing—original draft, Writing—review and editing. NS: Conceptualization, Data curation, Formal analysis, Resources, Software, Supervision, Validation, Writing—original draft. GW: Investigation, Supervision, Validation, Visualization, Writing—original draft.
